# Promoting Diversity through an Understanding of Barriers and Drivers for Inclusive Clinical Trials

**DOI:** 10.1007/s43441-025-00751-9

**Published:** 2025-02-26

**Authors:** Annick de Bruin, Jasmine Masullo, Shalome Sine, Kenneth Getz

**Affiliations:** 1 The Center for Information and Study on Clinical Research Participation, Boston, MA USA; 2 Tufts Center for the Study of Drug Development, Tufts University School of Medicine, Boston, MA USA

## Abstract

**Importance:**

Racially and ethnically diverse, equitable representation among clinical trial participants is important for enhancing the drug development process and promoting equitable healthcare outcomes.

**Objective:**

To understand the barriers and drivers for inclusive clinical trials, focusing on the attitudes, perceptions, experiences, and challenges faced by underrepresented populations.

**Design:**

An online questionnaire was administered online from April to June 2023 and involved 12,017 respondents from 54 countries. This survey utilized a convenience sampling strategy. Statistical analysis was performed to compare responses among racial and ethnic groups.

**Setting:**

The study was conducted globally. Survey respondents were recruited through various patient recruitment organizations, patient advocacy groups, and contract research organizations.

**Respondents:**

Adults 18 years or older who received an email or had online access were eligible to participate. Racial and ethnic composition included White (81%), Hispanic/Latino (15%), Black/African American (6%), Asian (6%), and other ethnicities.

**Exposure(s):**

Respondents were asked about their perceptions, concerns and experiences related to clinical research access and participation.

**Main Outcome(s) and Measure(s):**

Key outcomes included barriers to clinical research participation, factors influencing trust in pharmaceutical companies and past experiences.

**Results:**

Barriers to clinical research participation varied among ethnic groups. Asian respondents cited concerns about time off work (22%) and time required to participate (19%) more frequently as compared to White respondents (7% and 7%, respectively; *p* < 0.05). Hispanics expressed higher concerns about time off work (15%) and receiving placebo (10%) as compared to Non-Hispanics (8% and 5%, respectively, *p* < 0.05). Black and Hispanic respondents placed higher importance on diversity in staff compared to White and non-Hispanic respondents (B: 32%; W: 12%; Hispanic: 22%; Non-Hispanic: 13% *p* < 0.05). Black, Asian, and Hispanic respondents reported higher levels of disruption in participation related to technology use (Black: 31%; Hispanic: 30%; Asian: 29%) and completing study requirements at home (Black: 32%; Hispanic: 30%; Asian: 26%) as compared to White (13%, 15%; *p* < 0.05%) and non-Hispanic respondents (14%, 17%; *p* < 0.05).

**Conclusions:**

The findings highlight the need to address barriers to diversity in clinical trials and improve trial experiences of underrepresented communities, facilitating design of more inclusive and patient-centered trials.

## Key Points

**Question:**

What are the key barriers and drivers influencing the participation of underrepresented populations in clinical trials?

**Findings:**

This study identified barriers and disruptions to clinical trial participation and experiences disproportionately affecting those who identified as Black or African American, as well as those identifying as Hispanic or Latino, based on survey findings from 12,017 individuals (representing 54 countries). Additionally, diversity within clinical trial staff emerged as an important factor in building trust.

**Meaning:**

These findings provide insights for improving diversity in clinical trials and enhancing the participation experiences of underrepresented communities and may help advance equitable drug development and healthcare outcomes.

## Introduction

Diverse, equitable representation in clinical research is essential for strengthening the generalizability of findings during treatment development [1–9] and more accurately translating study results into clinical practice [10, 11]. However, racial and ethnic groups who are medically underserved, including those who are Black or African American and those who are Hispanic or Latino, remain underrepresented in clinical trials [12–22]. Some of the reasons for this underrepresentation include differences in perceptions and awareness of clinical research among those from underrepresented communities, including lack of trust in the medical system due to historical injustices as well as patient experiences of medical mistreatment [23]. Other reasons for underrepresentation include barriers to participation, such as a lack of culturally relevant outreach and engagement, restrictive eligibility criteria, language barriers, and logistical concerns, which disproportionately affect those from underrepresented communities [24]. 

In recent years, there has been heightened awareness and acknowledgement within the drug development industry of the importance of diverse representation among clinical trial participants [25], and regulatory efforts have focused on identifying ways to mitigate known barriers to clinical trial participation for underrepresented populations. For example, the US Food and Drug Administration (FDA) released guidance in June of 2024 aiming to enhance equitable racial and ethnic representation in clinical research participation, namely through provisions of Diversity Plans for all Phase 3 clinical trials related to drugs and biologics [26–28]. 

However, despite industry interest in achieving equitable representation among clinical trial participants, challenges to achieving this goal persist, and racial and ethnic groups, including those who are Black or African American and those who are Hispanic or Latino, remain underrepresented among clinical trial participants [20]. More research is needed to further examine the attitudes, preferences, perceptions, and experiences of clinical trial participation among those from underrepresented communities in order to identify actionable strategies for addressing barriers to participation.

In this article, we present key findings from a 2023 global survey involving 12,017 respondents, examining key differences in the perceptions, barriers, and experiences of clinical research across racial and ethnic subgroups, and identifying opportunities to improve patient engagement and mitigate barriers to participation for underrepresented populations.

## Methods

The Center for Information and Study on Clinical Research Participation (CISCRP), an independent nonprofit organization in Boston, Massachusetts, conducts a biennial global online survey called the “Perceptions & Insights” (P&I) study that since 2013 has evaluated public and patient perceptions, motivations, and experiences in clinical research [10, 29]. This current 2023 longitudinal study follows the American Association for Public Opinion Research (AAPOR) reporting guideline. Implied consent was used in the study and respondents were informed of the objectives, risks and benefits of the survey and assured of anonymity. As an incentive, respondents were offered entry into a gift card draw of $10.00 (or local currency equivalent) on completion of the survey.

### Survey Methodology and Data Collection

Each biennial survey is written and developed by a cross-functional work group comprising patients, representatives from patient advocacy groups, pharmaceutical companies, investigative research sites, and contract research organizations. Organizations including Clariness, James Lind Care, Benchmark Research, and Rare Patient Voice collaborated with CISCRP to engage respondents using a convenience sampling strategy. These organizations reached out to their networks of patients and the general population and disseminated an online link for survey participation. The instrument was translated into Spanish, German, French, Chinese, and Japanese, and the survey responses were collected online between April and June 2023. Respondents selected from a list of options to self-identify their own race and ethnicity identity, or write-in a race or ethnicity not provided by the list, but they were not required to provide this information. The survey included questions on clinical research perceptions, awareness, and engagement preferences. Respondents who indicated that they had experience participating in clinical trials were asked a series of additional questions specifically related to their participation experience. Longitudinal analyses were enabled by reusing some of the questions from the prior P&I studies, while incorporating new topics of heightened recent interest. These included some additional questions concerning respondents’ clinical research experiences with decentralized clinical trial elements, perceptions of alternative site settings such as pharmacies, and the obstacles they may face in trial participation.

### Statistical Analysis

The 2023 survey was programmed in and administered via an online survey tool. The 2023 data set was subsequently exported from the online survey tool and imported into data analysis and visualization software (MarketSight LLC). All questions were required in this survey, there was no missing data. Comparisons among ethnic subgroups in the study were performed using z tests, and comparisons of means were performed using t tests. Tests were conducted at the 95% confidence level. All tests were 2-sided, with *p* < 0.05 considered to be statistically significant. Potential biases related to sampling and methods used can be found in the Limitations section. The data for this article were specifically chosen to reveal statistically significant differences in patient perceptions and factors that might impact clinical trial participation, perceptions and experiences across racial and ethnic demographic groups.

### Ethical Review

The 2023 study was reviewed and deemed to be exempt by a central IRB - the WCG Institutional Review Board. All data were de-identified.

## Results

### Demographic Information

Table 1 summarizes the 2023 survey respondent characteristics. The online survey was distributed and completed globally (54 countries) by 12,017 respondents (from an estimated initial 30,000 contacted respondents) ages 18 and above. In the current survey, approximately two-thirds of respondents (61%) identified as female, 37% were male and 2% self-identified as other genders. The respondent sample was predominantly concentrated within the two major geographical regions of North America and Europe, with some participation from Asia Pacific, South America and Africa. The majority of survey respondents (81%) identified as White (9,418 respondents), while 6% of respondents identified as Black/African American (644 respondents) and another 6% identified as Asian (727 respondents). Most (85%) identified as non-Hispanic or Latino, with 15% of the respondents indicating Hispanic or Latino ethnicity (1,796 respondents),

**Table 1:** Respondent Profiles^a^

Characteristic	No. Respondents (%)
*Sample size*	12,017
Sex	
Male	4,488 (37%)
Female	7,333 (61%)
All other gender identities	196 (2%)
Region	
North America	5,605 (37%)
South America	273 (2%)
Europe	5,501 (46%)
Asia Pacific	504 (4%)
Africa	134 (1%)
Age	
18–34	2,234 (19%)
35–44	2,110 (18%)
45–54	2,199 (18%)
55–64	2,558 (21%)
≥ 65	2,916 (24%)
Race (top mentions)	
White	9,418 (81%)
Black or African American	644 (6%)
Asian	727 (6%)
Ethnicity (top mentions)	
Non-Hispanic/Latino	9,869 (85%)
Hispanic/Latino	1,796 (15%)
Educational level	
No school or primary	218 (2%)
Some or completed high school	2,242 (19%)
Some or completed college	7,451 (62%)
Completed postgraduate work	2,106 (18%)

^a^ Based on data from the 2023 Center for Information & Study on Clinical Research Participation Perceptions & Insights Study. Excludes “Prefer not to answer.”

### Recent Improvements in Perceptions of Safety and Willingness to Participate

In the years since the P&I study began, perceptions of the safety of clinical research have improved among respondents. In 2015, 26% of White respondents and 27% of Black or African American respondents indicated that they believed clinical research studies to be “very safe,” while in the 2023 survey, the proportion of those indicating “very safe” rose to 37% of White respondents and 41% of those who are Black or African American (data not shown).

Similarly, willingness to participate in a clinical trial has also increased among patients and the public. In 2015, 45% of those who are Black or African American and 40% of those who are White indicated that they would be “very willing” to participate in a clinical trial; in 2023, the proportion of those indicating that they would be “very willing” rose by 14% points to 59% of Black or African American respondents and by 10% points to 50% of White respondents (data not shown).

### Barriers to Clinical Research Participation

Though perceptions of safety have improved and respondents demonstrate a greater willingness to participate, barriers to participation persist. Of all respondents, less than half (47%) had ever been asked to participate in a clinical research study, and likelihood of being asked to participate varied across demographics. For example, those who were non-Hispanic were more likely (49%) to have been asked to participate in a clinical research study compared to those who were Hispanic (37%; *p* < 0.05, data not shown).

Additionally, not all respondents who tried to participate in a clinical trial were able to do so. Among all those who tried to participate in clinical research but did not enroll, the most frequently reported reason for non-participation was the failure to meet the specified study eligibility criteria (Table 2). Among both Black or African American (B) and White (W) groups, other top reasons included difficulty in reaching the research center (W: 16%, B: 12%), the time required for participation (W: 7%, B: 11%), concerns about research study risks (W: 7%, B: 9%) and other, self identified concerns (Table 2). Asian respondents more frequently cited barriers related to taking time off work (22%) and that too much time will be required for participation (19%). Asian respondents also expressed more concern about privacy (10%) in clinical research compared to White respondents (3%, *p* < 0.05).

Significant differences were also seen across the Hispanic and non-Hispanic ethnicity subgroups (Table 2). Hispanic respondents were more concerned about affordability of time away from their jobs (15%) compared to non-Hispanic respondents (8%, *p* < 0.05). Hispanic respondents also showed more concern of the risk of receiving a placebo as compared to non-Hispanics (10% vs. 5%, respectively; *p* < 0.05). Concerns about research risks were higher among Hispanic respondents (12%) compared to non-Hispanics (7%; *p* < 0.05).

**Table 2:** Perceived barriers to Clinical Research Study participation (2023) ^a^

Reasons for non-participation	No. (%) of Respondents by Race/Ethnicity							
	Total	White	Black or African American	Asian	All other races	Total	Not Hispanic/Latino/Spanish Origin	Hispanic/Latino/Spanish Origin
*Sample Size*	*2*,*313*	*1*,*846*	*137*	*136*	*194*	*2*,*325*	*1*,*978*	*347*
*Total Mentions*	*3*,*693*	*2*,*855*	*213*	*290*	*335*	*3*,*703*	*3*,*048*	*655*
I did not qualify to participate (i.e., I did not meet the requirements for the clinical research study)	1,669 (72)	1,371 (74)^b^	101 (74) ^b^	69 (51) ^b^	128 (66)	1,678 (72)	1,471 (74)^c^	207 (60) ^c^
I could not afford the time away from my job	203 (9)	136 (7) ^b^	10 (7) ^b^	30 (22) ^b^	27 (14)	203 (9)	151 (8) ^c^	52 (15) ^c^
I have concerns about the risks associated with clinical research studies	175 (8)	127 (7) ^b^	13 (9)	20 (15) ^b^	15 (8)	172 (7)	132 (7) ^c^	40 (12) ^c^
I do not want to take a chance with my health	147 (6)	106 (6)	9 (7)	17 (13)	15 (8)	146 (6)	120 (6)	26 (7)
It costs too much money to participate	122 (5)	96 (5)	8 (6)	10 (7)	8 (4)	124 (5)	96 (5)	28 (8)
I do not want to risk getting a placebo (i.e., inactive substance/sugar pill)	137 (6)	104 (6)	3 (2)	15 (11)	15 (8)	141 (6)	106 (5) ^c^	35 (10) ^c^
It is too difficult for me to get to the research center	361 (16)	291 (16)	16 (12)	29 (21)	25 (13)	361 (16)	313 (16)	48 (14)
Too much time is required to participate	189 (8)	129 (7) ^b^	15 (11)	26 (19) ^b^	19 (10)	187 (8)	147 (7)	40 (12)
Online information convinced me not to participate	56 (2)	33 (2) ^b^	5 (4)	7 (5)	11 (6) ^b^	58 (2)	36 (2) ^c^	22 (6) ^c^
My family/caregiver did not want me to participate	69 (3)	52 (3)	1 (1)	9 (7)	7 (4)	69 (3)	44 (2) ^c^	25 (7) ^c^
My doctor does not recommend participation in clinical research studies	68 (3)	48 (3)	3 (2)	7 (5)	10 (5)	67 (3)	49 (2)	18 (5)
My health insurance does not cover the costs	107 (5)	72 (4) ^b^	5 (4)	15 (11) ^b^	15 (8)	108 (5)	77 (4) ^c^	31 (9) ^c^
I am concerned about protecting my privacy	79 (3)	49 (3) ^b^	9 (7)	14 (10) ^b^	7 (4)	79 (3)	56 (3) ^c^	23 (7) ^c^
I have heard too many negative stories in the media and by word-of-mouth	54 (2)	27 (1) ^b^	6 (4)	10 (7) ^b^	11 (6) ^b^	56 (2)	33 (2) ^c^	23 (7) ^c^
Other - Write In (Required)	257 (11)	214 (12)	9 (7)	12 (9)	22 (11)	254 (11)	217 (11)	37 (11)

^a^ Based on the data from the 2023 Center for Information & Study on Clinical Research Participation Perceptions & Insights Study. The survey participants responses to the question “What was the primary reason you did not participate in the clinical research study?” Data included in this table is specific to individuals who tried to participate in clinical research but did not enroll (Non-Participant Filter: Have you ever participated in (i.e., joined or enrolled) a clinical research study? = No). Data based on all survey responses to the indicated question(s), excluding responses from participants who indicated “prefer not to answer” for race/ethnicity information

^b, c^ Indicates statistical significance at 95%CI compared with the other demographic group(s). The *z* test *P* value for age variable was corrected for type I error, and the test results were adjusted by multiplying the *P* value for each test by the *df*. Percentages (%) indicate % Valid Cases

### Factors that Influence Trust in Pharmaceutical Companies

Across the board, all respondents placed a high importance on the sponsoring pharmaceutical company sharing more information about the health risks and benefits of their medicines, with between 54% and 66% among various demographic groups indicating that this might increase their trust in the pharmaceutical companies conducting clinical trials (Table 3).

In the 2021 CISCRP survey, mistrust in pharmaceutical companies was cited as a reason for perceiving clinical studies as unsafe by 26% of Black, 41% of White, and 29% of Asian respondents. Notably, the 2023 study shows a significant improvement in trust, with only 12%, 14%, and 13% of Black, White, and Asian respondents, respectively, maintaining this view.

When citing measures that can help increase their trust, Black respondents placed a higher priority on the company including a diverse set of both participants and staff in their clinical trials (diverse participants: 52% of responses; diverse staff: 32% of responses), and these outcomes were also similar to the prior 2021 study (diverse participants; 51%; diverse staff: 37% among Black survey respondents). Black respondents also placed a higher emphasis on the company partnering with local community leaders and organizations, as compared to White respondents (B: 24%; W: 13%, *p* < 0.05). Hispanic respondents’ trust in pharmaceutical companies was similarly more likely to be increased by knowing that the company employed staff that was diverse (Hispanics: 22%; non-Hispanics: 13%; *p* < 0.05; Table 3).

Additionally, knowing that the company actively engaged with patients and caregivers to make clinical research more accessible was important for building trust among White (53%) and Black (51%) respondents as well as respondents of all other races (51%), but significantly less important for Asian respondents (35%, *p* < 0.05).

**Table 3:** Ways to Increase Perception of Trust in Pharmaceutical companies (2023) ^a^

Trust building factors	No. (%) of Respondents by Race/Ethnicity							
	Total	White	Black or African American	Asian	All other races	Total	Not Hispanic/Latino/Spanish Origin	Hispanic/Latino/Spanish Origin
*Sample Size*	*11*,*664*	*9*,*418*	*644*	*727*	*875*	*11*,*665*	*9*,*869*	*1*,*796*
*Total Mentions*	*41*,*226*	*33*,*175*	*2*,*527*	*2*,*378*	*3*,*146*	*41*,*255*	*35*,*011*	*6*,*244*
Knowing that the company employed staff that was diverse (e.g., mix of races, ethnicities, ages, genders, sexual orientations, etc.)	1,700 (15)	1,152 (12)^b^	204 (32)^b^	160 (22)^b^	184 (21)^b^	1,697 (15)	1,297 (13)^c^	400 (22)^c^
By the company sharing more information about the health risks and benefits of their medicines	7,470 (64)	6,193 (66)^b^	375 (58)^b^	396 (54)^b^	506 (58)^b^	7,483 (64)	6,438 (65)^c^	1,045 (58)^c^
By the company sharing more information about the drug approval process for their medicine	4,834 (41)	3,883 (41)	291 (45)	300 (41)	360 (41)	4,856 (42)	4,119 (42)	737 (41)
By the company sharing more information about the clinical research that has already been done on their medicines	6,267 (54)	5,157 (55)^b^	317 (49)	329 (45)^b^	464 (53)	6,259 (54)	5,359 (54)^c^	900 (50)^c^
By the company sharing more information about their company in general and its mission	3,292 (28)	2,575 (27)^b^	225 (35)^b^	220 (30)	272 (31)	3,295 (28)	2,782 (28)	513 (29)
By knowing that the company included a diverse set of participants in their clinical trials (e.g., mix of races, ethnicities, ages, genders, sexual orientations, etc.)	3,680 (32)	2,780 (30)^b^	336 (52)^b^	254 (35)^b^	310 (35)^b^	3,687 (32)	3,063 (31)^c^	624 (35)^c^
By the company sharing information in patient-friendly language that I can easily understand	5,600 (48)	4,648 (49)^b^	278 (43)	273 (38)^b^	401 (46)^b^	5,600 (48)	4,871 (49)^c^	729 (41)^c^
Knowing that the company actively works with patients, caregivers and patient communities to make clinical research studies easier to participate in	6,025 (52)	4,991 (53)^b^	330 (51)^b^	258 (35)^b^	446 (51)^b^	6,034 (52)	5,147 (52)	887 (49)
By the company partnering with local community leaders and organizations	1,667 (14)	1,235 (13)^b^	152 (24)^b^	129 (18)^b^	151 (17)^b^	1,670 (14)	1,320 (13)^c^	350 (19)^c^
Other - Write In	263 (2)	225 (2)	3 (0)	10 (1)	25 (3)	262 (2)	236 (2)	26 (1)
Nothing would increase my trust	428 (4)	336 (4)^b^	16 (2)^b^	49 (7)^b^	27 (3)^b^	412 (4)	379 (4)^c^	33 (2)^c^

^a^ Based on the data from the 2023 Center for Information & Study on Clinical Research Participation Perceptions & Insights Study. The survey participants responses to the question “What, if anything, might increase your trust in pharmaceutical companies that are conducting clinical research studies?” Respondents were asked to select all response options that applied. Data based on all survey responses to the indicated question(s), excluding responses from participants who indicated “prefer not to answer” for race/ethnicity information

^b, c^ Indicates statistical significance at 95%CI compared with the other demographic group(s). The *z* test *P* value for age variable was corrected for type I error, and the test results were adjusted by multiplying the *P* value for each test by the *df*. Percentages (%) indicate % Valid Cases

### Considerations for Enrollment and Study Logistics

When considering the logistics of clinical research studies, several criteria were deemed highly important across all respondent groups when deciding to participate, including required medical procedures, costs and reimbursements, study center proximity, and duration of participation, with more than half of respondents indicating that each of these factors would be “very important” to their participation decision (data not shown). However, among those who have never participated in a clinical research study, (n = 7,459), 41% considered compensated time off from work as “very important,” while past participants in clinical studies (n = 4,558) less frequently shared this view (35%; data not shown). Similarly, 34% of respondents who had never participated in clinical research placed some value on home delivery of clinical study medicine, and 27% considered home or personal office study visits as “very important”, while such views were shared by 28% and 25% of respondents with prior clinical trial experience, respectively.

Significant differences in the perceived importance of these logistical aspects of clinical trials also emerged across racial and ethnic subgroups. Black respondents were more likely to report the following as being ‘Very important’ compared to all other races: location of research study center, length of participation, number of study visits, duration of study visits, medical procedures required, flexible visit scheduling, remote visits, and potential costs/reimbursements. Hispanic respondents were more likely to report the following as being ‘Very important’ compared to non-Hispanic respondents: study medicine delivered to home and if time off from work is compensated (data not shown).

Black and Asian respondents, along with other races, were more likely to speak with family/friends when considering clinical trial compared to White respondents (B: 21%, A: 20%, W: 13% *p* < 0.05; data not shown). Respondents from several racial and ethnic groups were also more likely than White respondents to consult a community leader when looking for ways to learn about a clinical trial (B: 11%; W: 5%; A: 9%; Other: 8%; *p* < 0.05; data not shown).

Respondents generally showed a lack of awareness and understanding of the costs and reimbursement systems typically associated with trial participation; 11% of all respondents selected ‘false’ when asked if all medical costs would be covered by the study and 40% selected ‘false’ when asked if out-of-pocket costs such as gas, parking are reimbursed by the clinical research study (data not shown). These findings were consistent across different racial and ethnic groups.

### Participant Experiences in Clinical Trials

Traveling to the study clinic was identified as the most disruptive aspect of clinical research participation for all respondents. Notable differences emerged, however, across racial and ethnic subgroups in terms of other factors disrupting participation. Respondents of all races other than White expressed a greater challenge in having to complete study requirements at home, such as completing questionnaires, compared to respondents identifying as White (B: 32%; Asian: 26%; Other: 34%; W: 15%; *p* < 0.05; Table 4). In terms of receiving care however, Black respondents were more likely to report being “very comfortable” receiving care at home, as well as at the local supermarket and the local pharmacy compared to all other races (data not shown).

Black, Asian and Hispanic respondents reported higher levels of disruption when required to use technology like smartphones or tablets, compared to White and non-Hispanic respondents (W: 13%; B: 31%; Asian: 29%; *p* < 0.05; Hispanic: 30% vs. non-Hispanic: 14%; *p* < 0.05). Concerns about missing work or not getting paid were higher among those of All Other Races as compared to White (Other: 42% vs. W: 25%; *p* < 0.05). Interestingly, Hispanic respondents expressed less concern about traveling to the clinic (44%) as compared to non-Hispanics (59%; *p* < 0.05) and expressed more apprehension about having to complete study requirements at home (Hispanic: 30% vs. non-Hispanic: 17%; *p* < 0.05; Table 4).

When considering factors that can enrich the clinical trial experience, Black survey respondents indicated a higher likelihood of finding gestures of appreciation as helpful compared to White respondents. These included: thank you cards (B: 63%; W: 35%; *p* < 0.05), holiday cards (B: 74%; W: 37%; *p* < 0.05), items to support participation such as blankets, notebooks, or water bottles (B: 59%; W: 37%; *p* < 0.05), an invitation to partake in a satisfaction survey regarding their experience (B: 69%; W: 36%; *p* < 0.05) and certificates to commemorate their participation (B: 89%; W: 45%; *p* < 0.05; data not shown).

**Table 4:** Factors contributing to disruptive participation in Clinical Research studies (2023) ^a^

Factors that disrupt trial participation	No. (%) of Respondents by Race/Ethnicity							
	Total	White	Black or African American	Asian	All other races	Total	Not Hispanic/ Latino/ Spanish Origin	Hispanic/Latino/Spanish Origin
***Sample Size***	*777*	*537*	*62*	*102*	*76*	*780*	*618*	*162*
***Total Mentions***	*1*,*304*	*868*	*113*	*180*	*143*	*1*,*301*	*1*,*021*	*280*
Having to travel to the study clinic	443 (57)	330 (61)	30 (48)	47 (46)	36 (47)	438 (56)	367 (59) ^c^	71 (44) ^c^
Having to complete study requirements at home (such as completing questionnaires)	151 (19)	78 (15) ^b^	20 (32) ^b^	27 (26) ^b^	26 (34) ^b^	155 (20)	107 (17) ^c^	48 (30) ^c^
Too much time required	242 (31)	165 (31)	19 (31)	33 (32)	25 (33)	244 (31)	190 (31)	54 (33)
Having to use technology (such as a smartphone, tablet, etc.)	132 (17)	68 (13) ^b^	19 (31) ^b^	30 (29) ^b^	15 (20)	134 (17)	86 (14) ^c^	48 (30) ^c^
Missing work and/or not getting paid	225 (29)	136 (25) ^b^	21 (34)	36 (35)	32 (42) ^b^	222 (28)	169 (27)	53 (33)
Other - Write In (Required)	111 (14)	91 (17)	4 (6)	7 (7)	9 (12)	108 (14)	102 (17) ^c^	6 (4) ^c^

^a^ Based on the data from the 2023 Center for Information & Study on Clinical Research Participation Perceptions & Insights Study. The survey participants responses to the question “What made your participation in the clinical research study disruptive?” Data based on all survey respondents who indicated that the study was disruptive to their general daily routine, excluding responses from participants who indicated “prefer not to answer” for race/ethnicity information

^b, c^ Indicates statistical significance at 95%CI compared with the other age group(s). The *z* test *P* value for age variable was corrected for type I error, and the test results were adjusted by multiplying the *P* value for each test by the *df*. Percentages (%) indicate % Valid Cases

## Discussion

Despite government and industry prioritization, clinical trial diversity remains low, with consistent underrepresentation of certain demographic groups [12, 13, 21, 30]. Since 2013, CISCRP has been conducting large-scale, international, biennial surveys to assess global trends and changes in patient perceptions, motivations, and experiences related to clinical research participation. This ongoing approach utilizes a consistent and robust methodology to track and analyze shifts in patient perspectives over time [29, 31]. 

Contrary to the notion that underrepresented populations are less willing to participate in clinical research, consistently equal or greater willingness to participate in trials was observed as compared to those who are White [32, 33]. Additionally, perceptions of the safety of clinical trials have improved in recent years. Several factors may have contributed to these changes in public perception, including heightened public awareness of the drug development process and clinical trials in the wake of the events of the COVID-19 pandemic [34], as well as the effects of recent education and engagement efforts in the drug development industry, especially those aimed toward improving diversity, equity, and inclusion in clinical trials [35]. 

Our study findings highlight that the most prevalent barriers, aside from meeting the study eligibility requirements, were primarily logistical in nature such as difficulties in reaching research centers, time constraints, and affordability of time away from work. Logistics of trial participation may indeed pose significant and disproportionate challenges for underrepresented communities, including transportation issues, childcare access, and difficulties taking time off from work [16]. Hybrid or remote clinical trial models may help mitigate these logistical burdens, but our results indicate that comfort levels with receiving care at home, completing study requirements at home or at local pharmacies, and using technological devices to complete questionnaires, vary across race and ethnicity demographics. These results highlight the need for sponsors and collaborators to support customized approaches to clinical trial participation logistics for underrepresented populations to achieve proportional representation and meet their diversity action plan goals.

Interestingly, we have observed a decrease in the reported daily routine disruptions in our P&I study from 2021, when 26% of clinical trial participants indicated that their participation was somewhat or very disruptive to their daily routine potentially as a result of the pandemic. However, when comparing our current 2023 study results to an earlier 2017 CISCRP survey from six years ago, the findings suggest a potential return to the pre-pandemic conditions, with 18% of respondents reporting their participation as somewhat or very disruptive in 2023 as compared to 19% in 2017.

Previous studies have further reported that approximately one-third of respondents express mistrust towards pharmaceutical manufacturers, and medical mistrust tends to be more prevalent in underrepresented communities [16, 36–38]. To potentially increase their level of trust in pharmaceutical companies, Black respondents especially prioritized diversity in both trial participants and site staff. Those identifying as Hispanic, Asian, and All Other races also valued clinical trials with diverse staff. Additionally, knowing that the company actively engaged patients and caregivers to make clinical research more accessible was identified as likely to build trust among respondents.

Respondents identifying as Black/African American, Asian, and All Other races were also more likely to speak with family and friends, and were more likely to consult a thought leader, when considering clinical trial participation as compared to White respondents. Indeed, engaging a trusted community leader (e.g. clergy, trusted healthcare provider) has been shown to mitigate skepticism about clinical research, and certain patient advocacy initiatives, such as educational programs and mentorship efforts, have helped address participation barriers among underserved groups [39–44]. Public and patient education, particularly when led by individuals with clinical trial experience or community leaders can be highly effective [37, 40, 45]. Additionally, those who are Black/African American indicated especially strongly positive responses when asked about items and services which might acknowledge and encourage their participation, including thank-you cards, holiday cards, certificates of participation, and other items. These findings reinforce the importance of connecting with clinical trial participants on a personal level and acknowledging their contributions to the drug development process. Overall, findings indicate that gestures of appreciation, along with considerate and continuous community engagement, may be able to significantly impact the extent to which underrepresented populations feel included and valued during their clinical trial participation experience.

## Limitations

The results of the 2023 CISCRP survey, along with those from previous surveys, rely on responses obtained from convenience samples. While the study gathered a substantial number of responses (12,017 respondents), it is important to note that the surveys were conducted online among adults who self-identify as individuals already seeking health-related information, and who have chosen to receive email communications and invitations. Trends observed in CISCRP surveys also indicate a relatively stable pattern of ethnic representation over the years, however, mirroring clinical trial participation, there remains a limited number of voices from underrepresented populations contributing their input. Moreover, 80% of our respondents have stated that they had completed college, some college, and/or postgraduate work, suggesting that the respondent sample set was also well-educated. Therefore, it is essential to approach the survey findings with caution, as they may reflect sampling bias and might not accurately represent the perspectives of the entire global population, especially those who cannot access, receive, and read online solicitations and communications. Survey respondents answered questions based on their lived experience. Future publications are planned which will utilize the current study to explore other topics of global subgroup differences, age-specific barriers, disease severity and health status, and factors influencing trial dropouts by various demographic subgroups.

## Conclusions

The findings of the 2023 CISCRP study provide valuable insights into clinical trial engagement and participation of underrepresented communities, and examine the recent attitudes, perceptions, barriers, and experiences of these communities regarding clinical research. Our findings help inform patient engagement strategies toward improving participation experiences, promoting diversity in trials and advancing the drug development process.

## Data Availability

No data sets are publicly available. Further information may be requested by contacting the corresponding author.
